# Sex differences in the utilization of drugs for COVID‐19 treatment among elderly residents in a sample of Italian nursing homes

**DOI:** 10.1002/pds.5420

**Published:** 2022-02-28

**Authors:** Andrea Spini, Giada Crescioli, Sandra Donnini, Marina Ziche, Francesca Collini, Fabrizio Gemmi, Gianni Virgili, Alfredo Vannacci, Ersilia Lucenteforte, Rosa Gini, Niccolò Lombardi, Giuseppe Roberto

**Affiliations:** ^1^ Department of Medicine, Surgery and Neuroscience University of Siena Siena Italy; ^2^ Service de Pharmacologie Médicale University of Bordeaux Bordeaux France; ^3^ Department of Neurosciences, Psychology, Drug Research and Child Health, Section of Pharmacology and Toxicology University of Florence Florence Italy; ^4^ Tuscan Regional Center of Pharmacovigilance Florence Italy; ^5^ Department of Life Sciences University of Siena Siena Italy; ^6^ Regional Health Agency of Tuscany Florence Italy; ^7^ Department of Clinical and Experimental Medicine University of Pisa Pisa Italy

**Keywords:** azithromycin, COVID‐19, drug utilization, elderly, hydroxychloroquine, low‐molecular weight heparins, nursing homes

## Abstract

**Purpose:**

To describe the use of hydroxychloroquine (HCQ), azithromycin (AZI) and low‐molecular‐weight‐heparins (LMWH) by sex among elderly residents in nursing homes (NHs) of Tuscany region (Italy) during the first pandemic peak.

**Methods:**

A population‐based drug utilization study was performed linking the Tuscany Administrative Databases (TAD) to the regional COVID‐19 registry. Among elderly patients (≥ 65 years old) registered in TAD on 1st December 2019, new users of HCQ, AZI and LMWH that started the treatment during NHs stay (i.e., no dispensing of the same drug during the previous 6 months) were respectively identified in each month of observation period (December 2019–May 2020). The percentage of subjects diagnosed with COVID‐19 (COVID‐19+) on, or before, the date of first dispensing of the drug of interest was described. Results were stratified by sex (male = M; female = F).

**Results:**

New users of HCQ, AZI and LMWH were 62, 300, and 1215, respectively. About 95% of HCQ new users received the drug in April (M = 20; F = 40) of which 11 of men (55%) and 25 of women (62%) were COVID‐19+; AZI new users in April (M = 32; F = 83) were two‐fold compared to February, of which 10 of men (31%) and 30 of women (36%) were COVID‐19+; LMWH new users showed a peak in April (M = 74; F = 142), when 31% of men and 34% of women were COVID‐19 + .

**Conclusions:**

New users showed a peak during the first pandemic wave and females were over two‐fold compared to men. HCQ was often prescribed in absence of COVID‐19 diagnosis. During future global emergencies drug prescriptions should better be monitored in frail populations, especially in case of drugs with uncertain efficacy and safety.

**Key Points**

- No evidence is available on the utilization of drugs reimbursed for the treatment of COVID‐19 in the Italian nursing homes during the emergency of first pandemic wave.
- The absolute monthly number of hydroxychloroquine (HCQ), azithromycin (AZI) and low molecular weight heparins (LMWH) new users per sex and SARS‐CoV‐2 infection positivity was described between December 2019 and May 2020.
- Female new users were about two‐fold compared to men.
- In concomitance of the first pandemic wave, the number of HCQ, AZI and LMWH new users showed a peak in April for both sexes.
- HCQ was often prescribed in absence of a COVID‐19 diagnosis.

**Plain Language Summary**

We described the use of hydroxychloroquine (HCQ), azithromycin (AZI) and low‐molecular‐weight‐heparins (LMWH) by sex among elderly residents in nursing homes (NHs) of Tuscany region (Italy) during the first pandemic peak. We also described the percentage of subjects diagnosed with COVID‐19 (COVID‐19+). Results were stratified by sex (male = M; female = F). New users of HCQ, AZI and LMWH were 62, 300, and 1215, respectively. About 95% of HCQ new users received the drug in April (M = 20; F = 40) of which 11 of men (55%) and 25 of women (62%) were COVID‐19+; AZI new users in April (M = 32; F = 83) were two‐fold compared to February, of which 10 of men (31%) and 30 of women (36%) were COVID‐19+; LMWH new users showed a peak in April (M = 74; F = 142), when 31% of men and 34% of women were COVID‐19 +. New users showed a peak during the first pandemic wave and females were over two‐fold compared to men. HCQ was often prescribed in absence of COVID‐19 diagnosis. During future global emergencies drug prescriptions should better be monitored in frail populations, especially in case of drugs with uncertain efficacy and safety.

## 1 INTRODUCTION

Patients staying at nursing homes (NHs) are non‐independent elderly or disabled adults. They are a clinically frail population which is usually underrepresented in clinical studies. Moreover, although COVID‐19 pandemic has shown a clear sex difference in health outcomes (more severe symptoms and higher mortality in men as compared to women), evidence on sex differences in drug utilization for COVID‐19 is still scarce.^1^ The aim of this study was to describe the use of hydroxychloroquine (HCQ), azithromycin (AZI) and low molecular weight heparins (LMWH) by sex among elderly residents in NHs of Tuscany region (Italy) during the first pandemic wave of SARS‐CoV‐2 (March–May 2020). These medications were admitted to reimbursement by the National Healthcare Service as possibly useful for treating COVID‐19. Since efficacy and safety of these drugs was still uncertain, generating evidence on their actual use in such a frail population is of paramount importance in the context of a global emergency.

## 2 METHODS

This is a descriptive, drug‐utilization study. The population‐based, Tuscany Administrative Databases, which collect information on drugs reimbursed by the National Healthcare Service to Tuscany residents (about 3.7 million inhabitants), were linked to the regional COVID‐19 registry.

All elderly patients with ≥65 years of age and registered in the Tuscany Administrative Databases on 1st December 2019 with at least 6 months of look‐back period were selected. Among these subjects, new users of HCQ, AZI and LMWH starting the treatment during NHs stay between 1st December 2019 and 30th May 2020 were identified. New users were defined as subjects with >1 dispensing during the month of interest and none in the previous 6 months. Notably, given the administrative nature of the data sources used for this study, the actual indication of drug use was not available.

Using the regional COVID‐19 registry, the percentage of subjects recorded as positive to COVID‐19 (COVID‐19+) before or at the date of starting the drug of interest was also described. Results were stratified by male (M) and female (F) sex.

## 3 RESULTS

Table 1 shows the number of new users stratified per age bands during the observation period. Overall, in NHs new users of HCQ were 62 (M = 21, F = 41), of AZI were 300 (M = 102, F = 198), and of LMWH were 1215 (M = 426; F = 789). Female sex was more frequently observed in the group of subjects aged ≥85 years for all drugs of interest.

**TABLE 1:** Age‐class distribution of new users of hydroxychloroquine, azithromycin, and low molecular weight heparins in the nursing homes of Tuscany between December 2019 and May 2020

	December 2019		January 2020		February 2020		March 2020		April 2020		May 2020	
	Male	Female	Male	Female	Male	Female	Male	Female	Male	Female	Male	Female
Hydroxychloroquine												
Number of new users	0	0	0	0	0	1	1	0	20	40	0	0
Age classes (years, *N* %)												
65–74	–	–	–	–	–	0	1 (100.0)	–	3 (15.0)	1 (2.5)	–	–
75–84	–	–	–	–	–	1 (100.0)	0	–	14 (70.0)	13 (32.5)	–	–
≥85	–	–	–	–	–	0	0	–	3 (15.0)	26 (65.0)	–	–
Azithromycin												
Number of new users	11	18	19	40	14	23	16	25	32	83	10	9
Age classes (years, *N* %)												
65–74	1 (9.1)	0 (0.0)	3 (15.8)	3 (7.5)	4 (28.6)	4 (17.4)	4 (25.0)	3 (12.0)	3 (9.4)	5 (6.0)	0	0 (0.0)
75–84	6 (54.5)	6 (33.3)	7 (36.8)	10 (25.0)	5 (35.7)	4 (17.4)	7 (43.8)	5 (20.0)	13 (40.6)	24 (28.9)	5 (50.0)	2 (22.2)
≥85	4 (36.4)	12 (66.7)	9 (47.4)	27 (67.5)	5 (35.7)	15 (65.2)	5 (31.2)	17 (68.0)	16 (50.0)	54 (65.1)	5 (50.0)	7 (77.8)
Low molecular weight heparins												
Number of new users	77	135	85	178	78	129	65	111	74	142	47	94
Age classes (years, *N* %)												
65–74	12 (15.6)	14 (10.4)	11 (12.9)	22 (12.4)	15 (19.2)	14 (10.9)	11 (16.9)	16 (14.4)	15 (20.3)	13 (9.2)	13 (27.7)	9 (9.6)
75–84	32 (41.6)	44 (32.6)	32 (37.6)	64 (36.0)	32 (41.0)	45 (34.9)	29 (44.6)	30 (27.0)	32 (43.2)	49 (34.5)	11 (23.4)	32 (34.0)
≥85	33 (42.9)	77 (57.0)	42 (49.4)	92 (51.7)	31 (39.7)	70 (54.3)	25 (38.5)	65 (58.6)	27 (36.5)	80 (56.3)	23 (48.9)	53 (56.4)

### 3.1 Hydroxychloroquine users

**FIGURE 1:**
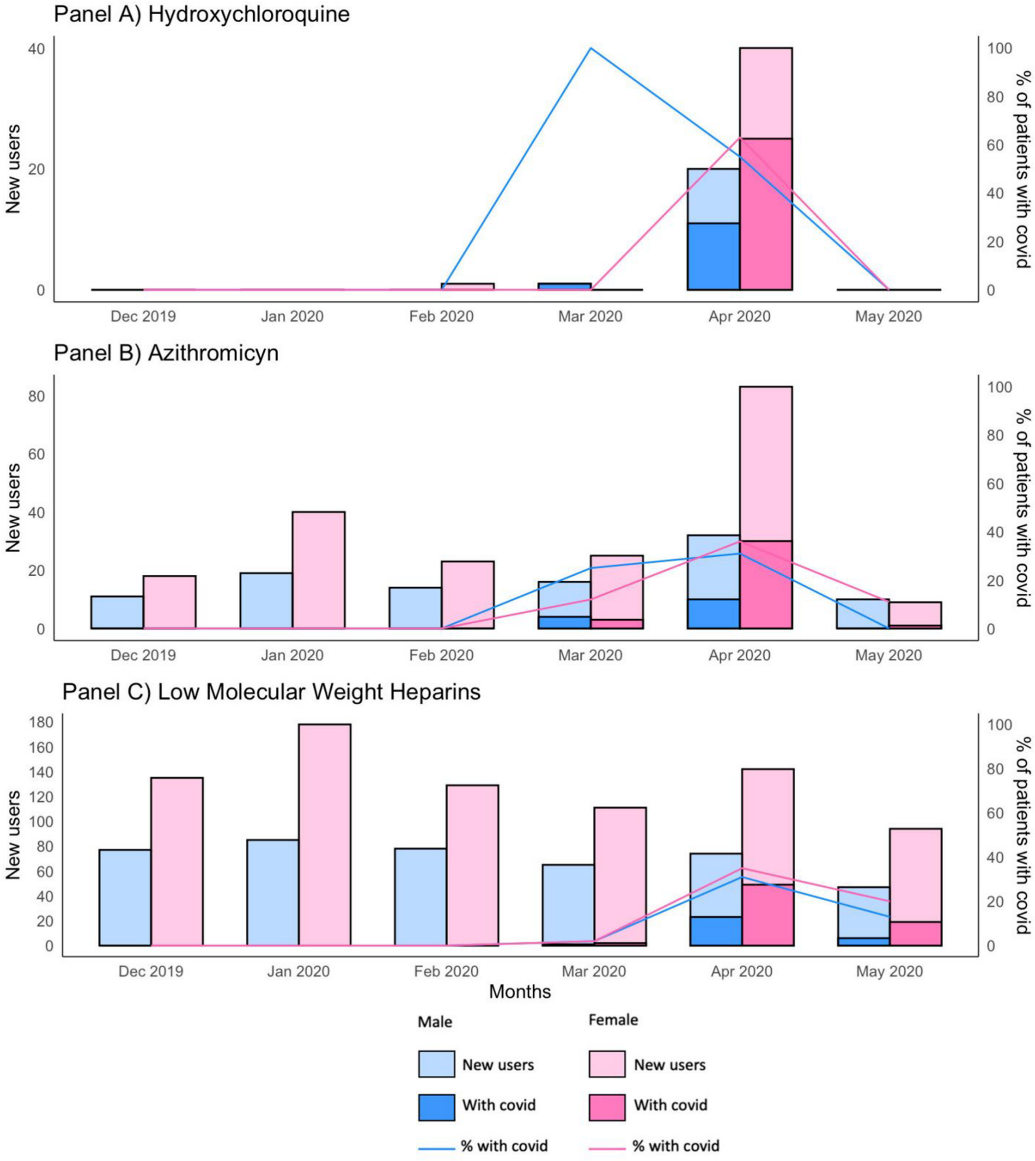
New users of HCQ (Panel A), AZI (Panel B) and LMWHs (Panel C) by sex and by positivity to COVID‐19 before start of the treatment

The 95% of new users of HCQ observed during the study period received the treatment during April 2020, while no new users of HCQ were observed outside the first pandemic wave, i.e., in the months of December, January and May (Figure 1, panel A). The percentage of COVID‐19+ new users at the time of the first dispensing in April 2020 were approximatively 60% for both sexes (M = 11/20; F = 25/40).

### 3.2 Azithromycin users

As reported in Figure 1 panel B, the number of AZI new users in the NHs rose markedly in April 2020. The number of new users increased from 14 and 23 in February 2020 to 32 and 83 in April 2020 for males and females, respectively. In April 2020, the percentage of COVID‐19+ new users at the time of first dispensing was below 40% in both sexes (M = 10/32; F = 30/83).

### 3.3 Low molecular weight heparins users

The number of new users of LMWH showed a peak in January 2020 (M = 85; F = 178) when no COVID‐19+ patients were observed. Afterword, LMWH new users showed a trend of decrease up to March 2020 (M = 65; F = 111) when COVID‐19+ users were only 3 (M = 1; F = 2). In April, the number of LMWH new users rose to a new peak of 74 in men and 142 in women, and the proportion of COVID‐19+ new users reached approximately 30% in both sexes (M = 23/74; F = 49/142) (Figure 1, panel C).

## 4 DISCUSSION

In this study, we observed that the number of new users of HCQ, AZI and LMWH in NHs in Tuscany showed a peak in April 2020, in concomitance with the peak of the first pandemic wave in Italy.^2^ The difference in age distribution between women and men staying in NHs^3^ is likely to explain the two‐fold higher number of female new users compared to men that was observed during the pandemic peak. With this respect, the observed number of women treated with HCQ, AZI and LMWH was about two‐fold compared to men, with a higher percentage of female patients aged ≥85 years.

As for HCQ, almost all new users identified in Tuscany NHs between December 2019 and May 2020 received the treatment in April 2020. Therefore, although the indication of drug use was not recorded, we can reasonably assume that all the residents received HCQ for COVID‐19 related issues. Unexpectedly, about 40% of them did not have a recorded diagnosis of COVID‐19 at the time of first dispensing. Thus, we cannot exclude that those NH residents received HCQ for COVID‐19 prophylaxis. Notably, the off‐label use of HCQ for the prevention of COVID‐19 was never allowed by the Italian Medicines Agency.^4^

Given the suspected efficacy of AZI in treating COVID‐19^5^ and the concomitant difficulty in quickly obtaining oropharyngeal swab results for adequate differential diagnosis, AZI was probably considered, in many cases, the antibiotic of choice for patients presenting with respiratory symptoms.

As for LMWH, the trend of decrease of the number of new users observed in February and March 2020 has to be mainly attributed to the discontinuation of all non‐urgent surgeries during the first pandemic wave. Nevertheless, in April 2020, the observed number of new users positive to SARS‐CoV‐2 rose markedly, particularly among women. Only 1 in 3 new users had a recorded COVID‐19 diagnosis, so the use of LMWH outside National recommendations, like treatment of suspected COVID‐19 cases or even prevention, cannot be excluded.

The main strength of this study was the record linkage between a large Regional healthcare database, the Administrative Database of Tuscany and the COVID‐19 registry. This allowed us to capture information from all NHs of the Tuscany region, thus increasing generalizability of results with respect to the population of elderly residents in Italian NHs. To the best of our knowledge, this is the first population‐based drug‐utilization study that describes the use of drugs admitted to reimbursement for the treatment of COVID‐19 in residents of NHs.

The most important limitation of this study is the lack of information on indication of HCQ, AZI and LMWH use. Indication for drug use, in fact, is not recorded in the database used for this study. Moreover, since previous studies reported that the first wave did not significantly affect the consumption of other drugs admitted for National Health System reimbursement^6^ (i.e., glucocorticoids, remdesivir, or vitamin D) they were not considered in this short analysis. Finally, we did not consider comorbidities and comedications that could have affected HCQ, AZI and LMWH use.

In conclusion, residents of NHs represent a particularly frail and old population. Their characteristics make them prone both to the occurrence of severe disease and death from SARS‐COV‐2, as well as to serious and potentially fatal adverse drug reactions. Although evaluating appropriateness of drug utilization using administrative databases is difficult, evidence from this study has to be intended as a signal of possible inappropriate prescribing that, particularly for HCQ, needs to be further investigated. In the context of a similar global emergency, prescribing behaviors in frail populations should be more strictly monitored, particularly when efficacy and safety of the prescribed drugs is still uncertain. Moreover, sex differences in drug utilization and consequent sex‐specific drug‐related issues should be carefully considered to ensure patients' safety.

## CONFLICT OF INTEREST

EL was involved as investigator of observational studies funded by the pharmaceutical company Galapagos in compliance with the ENCePP Code of Conduct, and she has carried out consultancy for Angelini.

## AUTHOR CONTRIBUTIONS

Study concept and design: GR, RG; Acquisition, analysis, or interpretation of the data: RG; Drafting of the manuscript: AS, GC, NL, GR; Supervision: SD, MZ, FC, FG, GV, AV, EL; Critical revision of the manuscript: All authors.

## ETHIC STATEMENT

The authors state that no ethical approval was needed.

## PREVIOUS PRESENTATIONS STATEMENT

This work was presented at the Annual Congress of the Italian Society of Pharmacology in February 2021 as an oral contribution.
